# Novel intramedullary device for lengthening transfemoral residual limbs

**DOI:** 10.1186/s13018-017-0553-8

**Published:** 2017-03-31

**Authors:** Todd A. Kuiken, Bennet A. Butler, Tom Sharkey, Andre D. Ivy, Daniel Li, Terrance D. Peabody

**Affiliations:** 1 Center for Bionic Medicine, Rehabilitation Institute of Chicago, 345 E. Superior St. Room 1309, Chicago, IL 60611 USA; 2 Departments of PM&R, Surgery, and Biomedical Engineering, Northwestern University, Chicago, IL 60611 USA; 3 Department of Orthopedic Surgery, Northwestern University Feinberg School of Medicine, 676 N. Saint Clair, Suite 1350, Chicago, IL 60611 USA; 4 DuPage Medical Group, 1801 South Highland Avenue Suite 220, Lombard, IL 60148 USA; 5 Northwestern University Feinberg School of Medicine, 420 E Superior St, Chicago, IL 60611 USA

**Keywords:** Amputation, Limb loss, Transfemoral amputation, Distraction osteogenesis, Limb lengthener

## Abstract

**Background:**

Lower limb loss is a highly disabling medical condition that can severely impact a person’s quality of life. Recovery becomes especially challenging if an amputee has a short residual limb, which can complicate proper prosthetic fitting, causing discomfort, difficulties in suspension, and reduced mobility. Current limb lengthening techniques such as the Ilizarov apparatus and external fixators are cumbersome, uncomfortable, and have high complication rates. In this study, we investigated the effectiveness of a novel limb-lengthening device that uses intramedullary bone lengthening and requires only one percutaneous rod at the end of the limb during the distraction phase. Only the intramedullary nail remains after the distraction phase, and no external components are required during the consolidation phase. We hypothesize that this system would create a much easier experience for the patient.

**Methods:**

The system was first tested in a mock surgical implantation using plastic femur bones. The device was then tested in a series of cadaveric experiments using pelvis-to-knee specimens by a group of surgeons. Surgeons evaluated the surgical insertion technique, soft tissue considerations, hardware fixation strategies, and the effectiveness of the distraction mechanism. Revisions and improvements to the device and surgical procedure were made based on the results from the cadaveric experiments.

**Results:**

A questionnaire was given to two visiting surgeons following the final iteration of the device. The surgeons reported that the system effectively lengthened the limb, was sturdy, and could be installed efficiently. However, there remains a risk of infection and soft tissue imbalances, similar to that introduced by an external fixator device. Suggestions on how to improve the design of the device and mitigate infection through postoperative management and surgical standard of care will be considered for future clinical trials.

**Conclusions:**

The described intramedullary residual limb-lengthening device has evolved from a prototype to a mature model tested in six cadaveric experiments to date. Further mechanical and functional testing is needed to finalize the device before testing in patients.

## Background

Limb loss is a devastating reality for millions of people worldwide. In the USA alone, there are an estimated 1.6 million amputees, a number that is expected to increase substantially by 2050 [1]. A significant number of these amputations are due to trauma, including 1645 combat injuries from the Iraq and Afghanistan wars that resulted in major limb loss, or oncologic disease, in which the exact amputation type and site are usually determined by the level of injury or pathology [2, 3]. In these cases, the residual limb length may be excessively short for ideal prosthesis fitting and utilization.

A short residual limb can be especially distressing for trauma or oncology patients, who are frequently young, but otherwise healthy, and have the potential for significant functional recovery through rehabilitation if fitted with a well-functioning prosthetic device [2]. In the case of excessively short transfemoral amputations (less than 35% of femoral length), it has been shown that a short residual limb can result in poor prosthetic fitting and wear, difficulty with adductor muscle balancing, and decreased hip strength. These complications combine to result in increased wound complications, increased energy consumption with ambulation, and decreased mobility [2, 4–11]. In addition, power transfer to the prosthesis is poor because the lever arm is short and encased in soft, compliant thigh tissue, thus compromising control of the prosthesis. With short transfemoral amputations, it is generally more preferable to lengthen the residual limb rather than to convert to a higher level of amputation given the major cosmetic and functional restraints associated with hip disarticulation [12].

Most current limb and/or residual limb lengthening methods rely on the process of distraction osteogenesis. First, an osteotomy is made and a distracting force is applied, typically with an external fixation device that also serves to stabilize the osteotomy site. After sufficient elongation has occurred, the newly generated bone is allowed to consolidate before removing the external fixator. While distraction osteogenesis is an option to improve skeletal deformities or treat complex fractures, the technique requires a lengthy process that is frequently associated with a number of complications, including pin site infection, nonunion, and malunion. Additionally, external fixation devices are generally cumbersome and uncomfortable for patients, often interfering with mobility, sleep, and the wearing of clothing [2, 10, 12–21]. The devices are also bulky, require 4–16 percutaneous wires or pins, and must be worn for a minimum of 3 days per millimeter of additional length (e.g., 300 days to gain 10 cm of length) [22].

Occasionally, an intramedullary device is used to augment lengthening and decrease the total time needed in an external fixation device. This strategy has been shown to have favorable results compared to external fixation-only methods [13, 15, 17, 18, 23–25]. While newer intramedullary nail-only lengthening techniques have been introduced for orthopedic surgeries, these are designed primarily for intact limb lengthening [26–28].

To address the need for easier limb lengthening systems in persons with above-knee amputation, our team at Northwestern University and the Rehabilitation Institute of Chicago (RIC) designed a novel residual limb-lengthening device that uses intramedullary (IM) bone lengthening and requires only one percutaneous component during the distraction phase. With only one distal percutaneous rod required and additional protective features designed to safely guide installation of the device, we hypothesize that this system will be as effective in limb lengthening as current external techniques but offer an easier, less burdensome option for patients, specifically for persons with transfemoral (above-knee) amputation.

## Methods

**Fig. 1:**
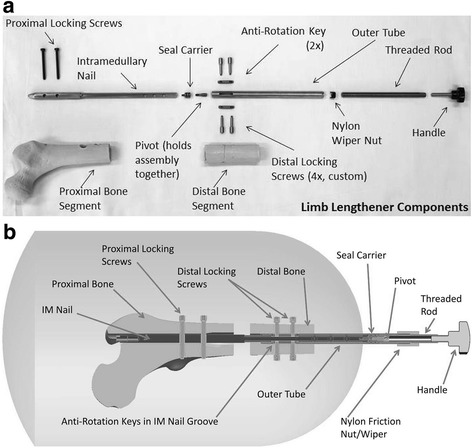
**a** Components of lengthening device disassembled. **b** Schematic of assembled lengthening device

The intramedullary (IM) lengthening device developed over the course of this study consists of three main components: an intramedullary (IM) nail, a threaded rod that abuts the distal end of the IM nail, and an extension tube with internal threading that fits over the IM nail/threaded rod construct [Fig. 1a, b]*.* Additional features include (i) a protective foam cover over the protruding parts of the system to provide safety and increased comfort, (ii) a double o-ring seal within the tube to act as an additional barrier and prevent bacteria from reaching the IM nail, (iii) two anti-rotation keys constrained to slide within a groove in the IM nail to prevent rotation between the two bone segments, and (iv) a targeting device to guide surgeons during the fixation process.

**Fig. 2:**
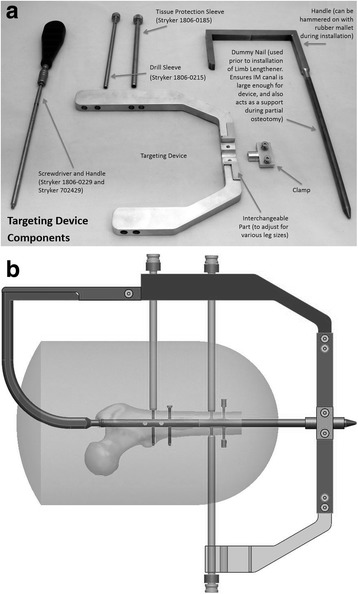
**a** Targeting guide components. **b** Targeting guide schematic (both anterograde and retrograde attachments shown)

For device insertion, the patient is placed in a supine position on a radiolucent table. Under C-arm guidance, a 1–2-cm incision is made at the distal end of the residual limb and blunt dissection is carried down to the distal end of the femur. If needed, a larger distal incision can be made to ensure that the muscular envelop of the residual limb is properly protected and that sites for myodesis are not damaged. Prior to performance of a transverse osteotomy, the distal femoral canal is opened (with an opening reamer if needed) and a stainless steel dummy nail is driven into the intramedullary canal of the femoral residual limb in a retrograde fashion. This confirms that the intramedullary canal is wide enough for proper insertion of the limb lengthener and provides a durable stabilizing post during the osteotomy itself. The osteotomy can be performed through a 2–3-cm direct lateral approach to the femur with a Gigli saw (passed around the femur with large right angle forceps with both handles exiting through the lateral incision). The osteotomy may then be completed if necessary with an osteotome. After osteotomy, the assembled lengthening device can be inserted into the intramedullary canal of the femoral residual limb in either an anterograde or retrograde fashion, using targeting guides developed specifically for the device (Fig. 2a, b).

**Fig. 3:**
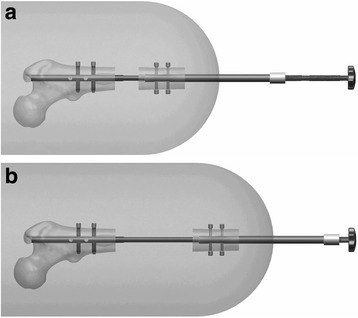
**a** Schematic of lengthening of residual limb using lengthening device. **b** Schematic of lengthening of residual limb using lengthening device

**Fig. 4:**
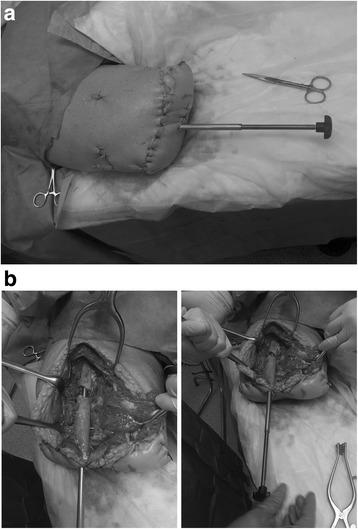
**a** Lengthening device implanted in minimally invasive, retrograde fashion into cadaveric specimen. **b** Demonstration of successful distraction at osteotomy site using lengthening device

The IM nail itself is fixed to the proximal bone segment with two interlocking screws (Stryker 1896-405OS) using static guide holes fixed into the targeting guide. The distal bone segment is attached to the extension tube with two screws bilaterally (up to four total) also using static guide holes fixed into the targeting guide. Once in place, the threaded rod can be rotated against the end of the fixed intramedullary nail to produce linear distraction of the extension tube and the attached distal bone segment (Figs. 3a, b and 4a–c). Notably, the fixation screws placed in the extension tube (two screws bilaterally) do not cross the entire construct. Instead, they penetrate the femoral cortex and thread through the near side of the extension tube before fixing themselves into an anti-rotation key. These keys (one per side) fit into longitudinal grooves in the side of the IM nail, allowing linear movement of the extension tube with respect to the IM nail but not rotation.

Following implantation of the device, a handle is attached to the distal end of the threaded rod. Rotation of this handle rotates the threaded rod, producing predictable longitudinal distraction of the extension tube and its attached bone segment with respect to the IM nail. Once the residual limb has been sufficiently lengthened, the handle, threaded rod, and extension tube may be removed. The remaining intramedullary nail is entirely contained in the limb.

In order to test the functionality of the installation guide, the device was initially tested in a mock surgical implantation using plastic femur bones. The device was then tested in a series of cadaver experiments using pelvis-to-knee specimens, in order to evaluate the surgical insertion techniques, how it interacted within the soft tissues, hardware fixation strategies, and the effectiveness of the distraction mechanism. During each trial, a prototype of the limb-lengthening device was implanted into a single cadaveric leg on which transfemoral amputation had been performed to create a short residual limb. In the first three sets of trials, a single cadaver was used; in the fourth trial, three cadaveric specimens were used. Refinements in the device were made after each test, and the improved version was then tested on a cadaveric specimen. Based on the outcomes of each trial, the device and its targeting guide underwent modifications and enhancements to improve the ease of implantation and its overall effectiveness. Below, we highlight important changes made to the device after each trial.

### Trial 1

- During insertion, the targeting device easily detached from the nail, resulting in problems with interlock targeting. We developed a stronger clamping mechanism to maintain alignment between the targeting device and limb lengthener.
- During drilling, the small screw holes in the extension tube were easily occluded by bone chips and soft tissue. We added a flat-tipped bottoming drill and irrigation to clear these screw holes after the initial drilling.
- We made the device thicker to increase its stiffness and durability, which decreases the chance of poor targeting of the drill sleeve during drilling.

### Trial 2

- The proximal interlocking screws were reduced in size from 5.0 to 4.0 mm (with a corresponding decrease in drill diameter from 4.2 to 3.5 mm) to improve the ease of targeting/insertion.
- The drill size used for the distal screws was increased to 5.0 from 4.2 mm to better uncover the distal screw holes and clear them of bony/soft tissue debris.

### Trial 3

- It was noted that a Gigli saw could cut through the aluminum dummy nail placed in the residual limb during the osteotomy. The dummy nail was replaced with a 440c stainless steel nail to increase its durability.
- Interchangeable arms were also added to the targeting device to accommodate wider residual limbs.

### Trial 4

- The gap between the proximal interlocking screws for the intramedullary nail and the distal fixation screws for the extension tube was decreased to allow for use on shorter residual femoral limbs.

## Results

**Fig. 5:**
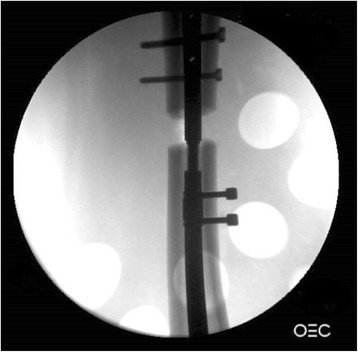
Radiographic demonstration of successful distraction at osteotomy site in cadaveric specimen using lengthening device (note: only unilateral distal fixation screws are placed in this case)

Insertion of the device into the cadaveric specimens took approximately 15–20 min for each surgeon. An X-ray image of the limb was taken to establish successful distraction at the osteotomy site in the cadaveric specimen using the lengthening device (Fig. 5). Following the trials, two members of the surgical team (visiting surgeons) were given a questionnaire to evaluate the device. They were asked questions on ease of installation, rigidity and strength of the device, and their impression of the design of the system. They were also asked to comment on any complications they anticipate during the patient’s use of the device and to discuss any changes or additional features they would like to see on the targeted device. All visiting surgeons reported that they “strongly agreed” that overall the limb lengthener was easy to install, was sturdy and rigid enough, and seemed to be strong enough for its intended use. The surgical team did not report any specific negative comments about device insertion. However, in discussing anticipated problems for use in patients, two major concerns arose that could occur during the distraction phase: (1) deep and superficial infections and (2) soft tissue imbalances (varus deformity) created during lengthening. These concerns are important to address with any percutaneous device. The team advised that these issues should be addressed through meticulous wound care and additional soft tissue balancing procedures as needed (See “Discussion”). Comments regarding potential complications during patient use of the device and desired additional features will be taken into account for future iterations of the device.

## Discussion

Most current limb lengthening techniques employ cumbersome devices and are fraught with high rates of complications. Intramedullary nail lengthening techniques with or without external fixator support have been shown to increase patient comfort relative to traditional all-external limb lengthening techniques [4, 10, 13, 15, 17, 23–25]. Currently available devices using these techniques include the Albizzia nail, the ISKD® (Intramedullary Skeletal Kinetic Distractor) system (Orthofix Holdings, Inc. Lewisville, TX), and the FITBONE® intramedullary nail (WITTENSTEIN intens GmbH, Germany). These devices have been shown to reduce time spent with an external fixator (during the consolidation phase of lengthening), reduce rates of infection, and allow for faster rehabilitation [26–28]. However, these systems are primarily designed and used for lengthening intact limbs [17, 26, 27].

Amputation enables a very different and much easier way to lengthen the residual limb long bone, as surgeons can easily access the end of the bone. Yet, to our knowledge, there have been no devices designed specifically for use as residual limb lengtheners. Most of the literature on the topic of residual limb lengthening comes from case reports or small case series. These describe either lengthening via distraction osteogenesis with an external fixation device or with vascularized bone flaps [6, 7, 10, 29–33]. Most authors have not only reported high rates of soft tissue complications and infection but also report reasonable improvements in prosthetic fitting and function [6, 10, 32]. An alternate strategy was reported recently by Henrichs et al., who replaced the entire proximal femur with a modular endoprosthesis in 28 oncology patients to avoid hip disarticulation; however, as with other strategies, this resulted in a relatively high rate of infection and soft tissue complications [34]. Of note, there is a recent case report by Paulsen et al. describing residual limb lengthening over a nail. Their group used the FITBONE® device, which is designed for lengthening intact limbs, to lengthen a transfemoral amputation. It is unclear what, if any, modifications had to be made to the device to allow for its use on a residual limb [35].

This study documents the development of the first known device designed specifically to lengthen transfemoral amputation residual limbs for the purpose of improving prosthetic fitting, prosthesis comfort, and ambulation. The device (i) has only one distal percutaneous component, (ii) allows accurate control of lengthening, and (iii) stabilizes the residual bone during consolidation, for earlier weight bearing. We hypothesize that compared to external fixators, such a device could be less cumbersome and possibly result in fewer complications for patients during the lengthening process. As noted above, there is a report of residual limb lengthening using a femoral lengthening nail (FITBONE®). Although this is a potentially attractive option, at present, such a nail would need to be custom ordered and likely manufactured on a case-by-case basis. This is an expensive option that would likely be an option only for patients with adequate insurance coverage who are treated at a large medical center. In comparison, our device is designed to be useful in a wide-range of clinical scenarios. It is probable that the simple construction of our device would allow for significantly decreased cost compared to all internal nails (which require somewhat complex mechanisms for lengthening). At the very least, this device could serve as a useful addition to a surgeon’s armamentarium for dealing with short transfemoral residual limbs.

Early results in cadaveric models indicate that the device can be implanted quickly and easily by surgeons. Its reliable targeting guide allows it to be placed in a minimally invasive fashion without the need for significant fluoroscopy or substantial experience with the device. Following implantation, the device provides consistent lengthening with the use of its handle. Finally, the lengthening components of the device are easily removed, once again in a minimally invasive fashion without a significant need for fluoroscopy, once sufficient lengthening is achieved.

Though this device has been successfully implanted in cadaveric models, it is important to note that it has yet to be tested on patients. Many aspects of the device’s in vivo performance, such as lengthening ability, mechanical integrity, local soft tissue response, and patient satisfaction can only be predicted based on previous work with analogous limb lengthening techniques [19, 24, 26–28]. There is a risk for both superficial and deep tissue infections to be introduced through the percutaneous attachment of the threaded rod/handle, similar to current external fixator lengthening techniques. Although the percutaneous attachment on our device is slightly larger than standard external fixator pins, our device should not introduce further skin/wound deformation with longitudinal pin migration as is seen with external fixator lengthening. Meticulous wound care at the insertion site, and possibly, prophylactic antibiotics would be needed to combat infection risk. If a clinically significant infection were to occur, the device could be removed relatively easily through its percutaneous attachment site (with fluoroscopic guidance to remove crossing screws).

Additionally, soft tissue balancing may be challenging during lengthening with this device, as it is with many lengthening techniques. The surgeon should anticipate a potential need for balancing procedures either at the time of surgery or at a later date. The exact procedures needed would have to be determined on an individualized basis depending on preoperative residual limb length/muscular attachments preserved, additional length desired, and the technique used for their original amputation.

Finally, the threaded rod emerging from the distal end of the residual limb could be an impediment during mobilization and a general irritant to patients. Even so, this single percutaneous element would likely be less cumbersome to patients than an external fixator, especially a ringed fixator. Theoretically, a prosthesis could even be designed with a small cutout to accommodate the distal end of the threaded rod (the handle would only need to be attached for rotation/lengthening). Furthermore, after lengthening, the threaded rod is removed, leaving only the IM rod in place during consolidation. In contrast, an external fixator would need to remain in place for the duration of the consolidation phase to prevent fracture or deformity. Our limb-lengthening device is currently undergoing approval testing with the FDA and will undergo further modifications as necessary before trials in patients begin.

## Conclusions

The cadaveric experiments and subsequent design iterations have led to a mature and functional device that has been evaluated in six cadaveric specimens to date. Further testing is needed in patients to determine if the device is safe, durable, and effective enough for its intended use of lengthening transfemoral residual limbs.

## Abbreviations

FDA: Food and Drug Administration
IM: Intramedullary
RIC: Rehabilitation Institute of Chicago
